# Verses

**Published:** 1907-11

**Authors:** 


### VERSES.

### TO MAN.

Here's to man—he is like a kerosene lamp: he is not especially bright; he is often turned down; he generally smokes; and he frequently goes out at night.

### A TOAST TO ALL.

Let us drink to the health of the bride,
Let us drink to the health of the groom;
Let us drink to the parson who tied,
And to every guest in the room.

### PASSING OF MONEY.

If money talks,
As some folks tell,
To most of us
It says: "Farewell!"

—Lippincots.

### GETTING A SEAT.

The interurban trolley car
The passengers called a cheat—
Too many were abroad by far,
And half were on their feet.
But the conductor had a pretty wit,
And his words were passing sweet,
For he promised that they all could sit
When they reached the country seat.

—Milwaukee Journal.

### PAT'S TOOTH PULLING.

Pat sat on a stool with a toothache rank,
The grinder tied to a door knob near,
And waited with patience the expected yank,
Tho' racked with pain and trembling with fear.

"Kape still me batin heart," he cried,
When he heard footsteps towards him hurrying.
"Swape me up," then next he sighed,
"And give me a dacent burying."

But when he found he'd see another morrow
He arose with spirit and talked quite civil,
He picked up the tooth and with a "begorrow,"
Wished "bad cess to the little aching divil."

B. H. Teague.

